# Structural basis of a microbial trimethylamine transporter

**DOI:** 10.1128/mbio.01914-24

**Published:** 2024-11-22

**Authors:** Chao Gao, Hai-Tao Ding, Kang Li, Hai-Yan Cao, Ning Wang, Zeng-Tian Gu, Qing Wang, Mei-Ling Sun, Xiu-Lan Chen, Yin Chen, Yu-Zhong Zhang, Hui-Hui Fu, Chun-Yang Li

**Affiliations:** 1 State Key Laboratory of Microbial Technology, Marine Biotechnology Research Center, Shandong University, Qingdao, China; 2 MOE Key Laboratory of Evolution and Marine Biodiversity, Frontiers Science Center for Deep Ocean Multispheres and Earth System & College of Marine Life Sciences, Ocean University of China, Qingdao, China; 3 Laboratory for Marine Biology and Biotechnology, Qingdao Marine Science and Technology Center, Qingdao, China; 4 Joint Research Center for Marine Microbial Science and Technology, Shandong University and Ocean University of China, Qingdao, China; 5 Antarctic Great Wall Ecology National Observation and Research Station, Polar Research Institute of China, Ministry of Natural Resources, Shanghai, China; 6 School of Life Sciences, University of Warwick, Coventry, United Kingdom; Case Western Reserve University School of Medicine, Cleveland, Ohio, USA

**Keywords:** TMA, TMA transporter, cryo-EM structure, binding mechanism, transport mechanism

## Abstract

Trimethylamine (TMA), a simple trace biogenic amine resulting from the decomposition of proteins and other macromolecules, is ubiquitous in nature. It is found in the human gut as well as in various terrestrial and marine ecosystems. While the role of TMA in promoting cardiovascular diseases and depolarizing olfactory sensory neurons in humans has only recently been explored, many microbes are well known for their ability to utilize TMA as a carbon, nitrogen, and energy source. Here, we report the first structure of a TMA transporter, TmaT, originally identified from a marine bacterium. TmaT is a member of the betaine-choline-carnitine transporter family, and we show that TmaT is an Na^+^/TMA symporter, which possessed high specificity and binding affinity toward TMA. Furthermore, the structures of TmaT and two TmaT-TMA complexes were solved by cryo-EM. TmaT forms a homotrimer structure in solution. Each TmaT monomer has 12 transmembrane helices, and the TMA transport channel is formed by a four-helix bundle. TMA can move between different aromatic boxes, which provides the structural basis of TmaT importing TMA. When TMA is bound in location I, residues Trp146, Trp151, Tyr154, and Trp326 form an aromatic box to accommodate TMA. Moreover, Met105 also plays an important role in the binding of TMA. When TMA is transferred to location II, it is bound in the aromatic box formed by Trp325, Trp326, and Trp329. Based on our results, we proposed the TMA transport mechanism by TmaT. This study provides novel insights into TMA transport across biological membranes.

**IMPORTANCE:**

The volatile trimethylamine (TMA) plays an important role in promoting cardiovascular diseases and depolarizing olfactory sensory neurons in humans and serves as a key nutrient source for a variety of ubiquitous marine microbes. While the TMA transporter TmaT has been identified from a marine bacterium, the structure of TmaT and the molecular mechanism involved in TMA transport remain unclear. In this study, we elucidated the high-resolution cryo-EM structures of TmaT and TmaT-TMA complexes and revealed the TMA binding and transport mechanisms by structural and biochemical analyses. The results advance our understanding of the TMA transport processes across biological membranes.

## INTRODUCTION

Biogenic trace amines are endogenous molecules produced as degradation products from macromolecules or fermentation of dietary amine-containing precursors (1–4). Though pioneering research on biogenic amines traditionally focused on their roles as neurotransmitters, it is now evident that certain unconventional biogenic amines play previously underestimated roles in both health and disease (1, 4–6). Of particular interest is the volatile trimethylamine (TMA), known for its pungent odor reminiscent of rotting fish (5, 6). While TMA has long been recognized for its involvement in the genetic disorder trimethylaminuria, recent studies have begun to unveil its presence in a range of human metabolism, including promoting cardiovascular diseases and acting as an agonist for human G protein-coupled trace amine-associated olfactory receptors (2, 3, 5–8). Arguably, however, TMA metabolism is best studied in microorganisms, being a precursor for the formation of the potent greenhouse gas methane by methanogenic *Archaea*, as well as a key nutrient source for some cosmopolitan marine microbes (9–12). What is more, TMA is also a precursor of trimethylamine *N*-oxide (TMAO), an important osmotic and hydrostatic pressure protectant used by many marine biota (13–18). Thus, in the deep-sea bacterium *Myroides profundi* D25, TMA is taken into the cells through a TMA transporter TmaT and then oxidized to TMAO, resulting in the accumulation of intracellular TMAO to millimolar levels to function as a piezolyte (13). This hydrostatic pressure tolerance strategy is widely adopted by marine Bacteroidetes (13).

Currently, two transport proteins have been implicated in TMA transportation across microbial membranes: MttP and TmaT (13, 19). MttP belongs to the drug/metabolite transporter superfamily (transporter classification number 2.A.7) (20), which predominantly consists of symporters and antiporters (21). MttP is located in the neighborhood of TMA methyltransferase involved in methanogenesis in several methylotrophic methanogens (19). It is hypothesized that MttP may transport TMA in these archaea though its function in TMA transportation remains to be validated. TmaT belongs to the betaine-choline-carnitine transporter (BCCT, transporter classification number 2.A.15) family (13, 22), and members of BCCT transporters are found in all three domains of life (22, 23). The BCCT family transporters are best known for transporting organic osmolytes into cells, fending off the detrimental effects of high osmolarity (22, 24, 25). The substrates of BCCT carriers often possess the feature with quaternary ammonium groups [R-N^+^(CH_3_)_3_], such as choline, *L*-carnitine, and glycine betaine (22, 24, 26, 27). Prototypes of BCCT transporters are energized by proton (BetT) or sodium gradients (BetP) (22, 24, 28, 29), whereas CaiT is an H^+^/Na^+^ independent antiporter (Table S1). TmaT was first identified in *M. profundi* D25, which only exhibits modest sequence identity to BetP (35%) from *Corynebacterium glutamicum* and CaiT (23%) from *Escherichia coli*, two members of the BCCT family with their crystal structures solved (13, 30, 31). In contrast to the putative TMA transporter MttP, our previous work has shown that TmaT can indeed bind TMA, and when expressed heterologously in *Escherichia coli*, TmaT enables TMA transport across the membrane (13). Phylogenetic analysis, however, indicates that TmaT forms a separate clade from the reported transporters BetP and CaiT, therefore representing a new member of the BCCT family (13). Thus, structural elucidation of TmaT is warranted to uncover the mechanism by which TMA is transported. Here, we report the first known structures of a TMA transporter TmaT and TmaT-TMA complexes using cryo-EM single-particle technique. Through a series of structural and biochemical analyses, together with molecular dynamics simulations, we propose the binding mode of TMA by TmaT and report the underlying transport mechanisms.

## RESULTS

### TmaT is a TMA-specific sodium-dependent transporter

**Fig 1:**
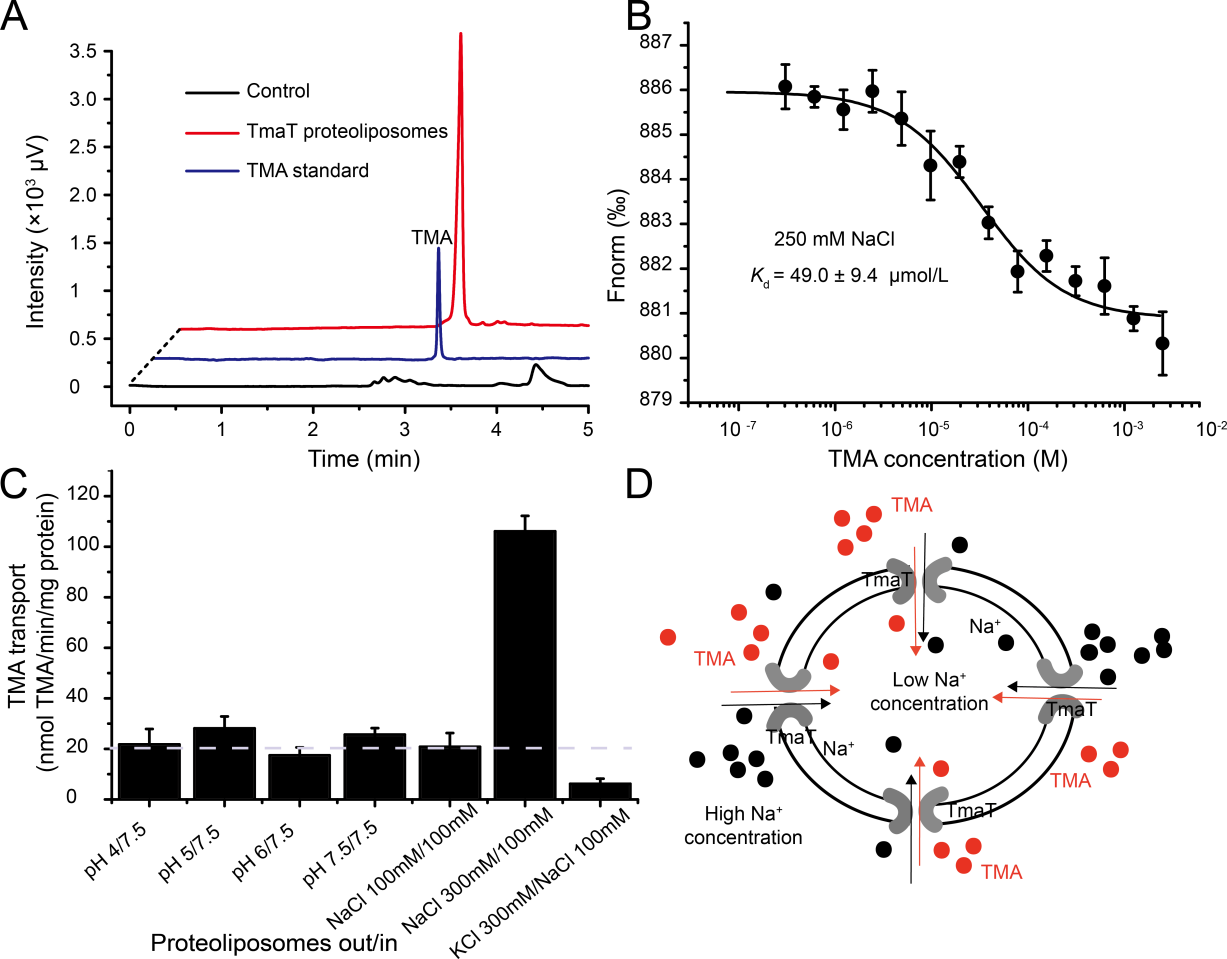
Detection of the TMA binding and transport activity of TmaT. (**A**) The TMA transport activity determined by gas chromatography with the TmaT proteoliposomes. The liposomes without TmaT were used as the control. The data shown are representatives of triplicate experiments. (**B**) Microscale thermophoresis analysis of the TMA binding activity of TmaT. The y-axis represents the normalized fluorescence (Fnorm). The error bars represent standard deviation of triplicate experiments. (**C**) The TMA transport activities with TmaT proteoliposomes under various pH and Na^+^ concentration conditions. The threshold line represents the average rate of transport with Na^+^ concentrations equivalent inside and outside the proteoliposomes at neutral pH. The error bars represent standard deviation of triplicate experiments. (**D**) A model of the transport of TMA and Na^+^ via TmaT proteoliposomes.

Full-length *tmaT* of *M. profundi* D25, which contains 1,590 nucleotides encoding a 529-amino-acid polypeptide, was overexpressed in *E. coli* C43(DE3), and the recombinant TmaT was purified (Fig. S1A). To ascertain whether the recombinant TmaT is able to transport TMA, the TmaT proteoliposomes were constructed. Transport of TMA mediated by TmaT proteoliposomes was measured by gas chromatography (GC), and the results suggested that the recombinant TmaT was a functional TMA transporter (Fig. 1A). The recombinant TmaT possessed a high binding affinity toward TMA, with a dissociation constant (*K*_*d*_) of 49.0 µM (Fig. 1B). Notably, the binding affinity of TmaT toward TMA was affected by NaCl concentrations. With 100 mM NaCl in solution, TmaT displayed a relatively lower binding affinity toward TMA (*K*_*d*_ = 227.6 µM, Fig. S1B), which is consistent with our previous work (13). With 500 mM NaCl, TmaT exhibited a relatively higher binding affinity toward TMA (*K*_*d*_ = 14.6 µM, Fig. S1B). However, no binding affinity of TmaT toward other tested quaternary amines, including glycine betaine, choline, carnitine, dimethylamine, or TMAO (Fig. S1C; Table 1), was observed with different NaCl concentrations, indicating that TmaT is a TMA-specific transporter.

**TABLE 1:** Analysis of the binding activities of TmaT toward TMA and its analogs^*a*^

Substrate	*K*_*d*_ (μM)
TMA	49.0 ± 9.4
Betaine	−
Choline	−
Carnitine	−
TMAO	−
Dimethylamine	−

^*a*^ The *K*_*d*_ value was determined by microscale thermophoresis at 22°C. The standard error is from three independent experiments. −, little binding activity was detected under the experimental conditions.

The majority of BCCT transporters are co-transporters that function alongside either hydrogen ions or sodium ions (22). To investigate the co-transport substrates of TmaT during TMA transport, we conducted experiments where TmaT proteoliposomes were incubated with TMA under various pH and Na^+^ concentration conditions. The results revealed that TmaT proteoliposomes displayed similarly low transport efficiencies toward TMA across different pH gradients. However, TmaT exhibited a much higher transport efficiency toward TMA under high Na^+^ concentrations outside the membrane (Fig. 1C). This suggests that TmaT is a sodium/TMA symporter (Fig. 1D), which is also consistent with the microscale thermophoresis (MST) analysis (Fig. S1B).

### Overall structure of TmaT

**Fig 2:**
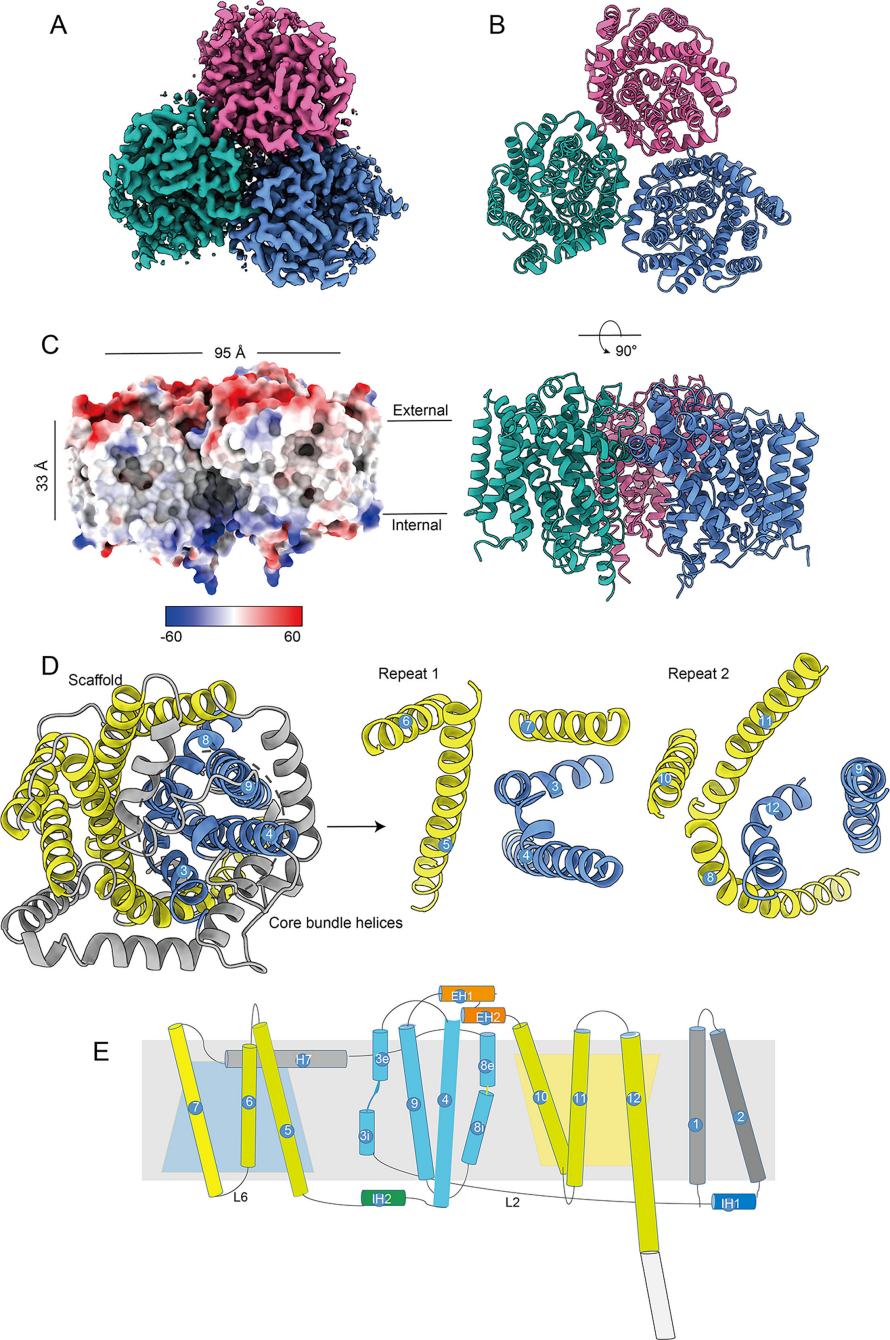
Overall structure analysis of TmaT. (**A**) Cryo-EM density maps of TmaT. (**B**) The overall structures of TmaT trimer with the top view and side view. Different monomers are colored in blue, red, and green, respectively. (**C**) Electrostatic surface representation of TmaT. (**D**) Top view of one TmaT monomer with two inverted repeats. (**E**) Schematic view of the topology of TmaT. The corresponding transmembrane helices in two inverted repeats are colored consistently. TM1–2 colored in gray, TM3–4 and TM8–9 in cyan, TM5–7 and TM10–12 in yellow, respectively.

To elucidate the mechanism of TmaT importing TMA, we employed the cryo-EM single-particle technique to determine the TmaT structure (Fig. S2; Table S2). TmaT assembles a homotrimer in solution (Fig. 2A and B), similar to other reported structures of BCCT carriers (30, 31). The overall structure of TmaT consists of three regions: the extracellular, intracellular, and transmembrane regions, with the overall transmembrane width of approximately 33 Å (Fig. 2C). The outer membrane region of TmaT is predominantly negatively charged, while the inner membrane region is mainly positively charged (Fig. 2C). The structures of the three TmaT monomers are nearly identical (with the root mean square deviation [RMSD] of 0.2–0.4 Å), with each monomer forms a cylindrical structure consisting of 12 transmembrane helices (TM1–TM12, Fig. 2D and E). Most of the transmembrane helices are connected by short loops, with the exception for TM7 and TM8, which are linked by a long helix (H7) (Fig. 2D and E). TM3 and TM8 consist of both inner membrane portions (TM3i and TM8i) and outer membrane portions (TM3e and TM8e), making them discontinuous transmembrane helices (Fig. 2E). TM3, TM4, TM8, and TM9 collectively form a four-helix bundle, while TM5–7 and TM10-12 form two scaffold-like structures that stabilize this bundle (Fig. 2D), which is similar to BetP (31). TmaT exhibits the typical two inverted repeat motifs of the five-transmembrane helix topology found in LeuT-type transporters (32, 33). In TmaT, TM3–7 corresponds to the first segment of the five-transmembrane helix (repeat 1), while TM8–12 corresponds to the second segment of the five-transmembrane helix (repeat 2). The two segments of five-transmembrane helices exhibit reverse symmetry (Fig. 2D and E). In the TmaT trimer structure, the loop Thr304-Gly313 from one monomer inserts into an adjacent monomer and resembles a gate latch and bolt pattern (Fig. S3). In addition, hydrogen bond interactions are formed with the residues Glu290, Asn291, Thr308, and Tyr309 in the interface between two adjacent monomers, suggesting a relatively stable trimer composition (Fig. S3).

### Different conformations of TmaT in binding TMA

**Fig 3:**
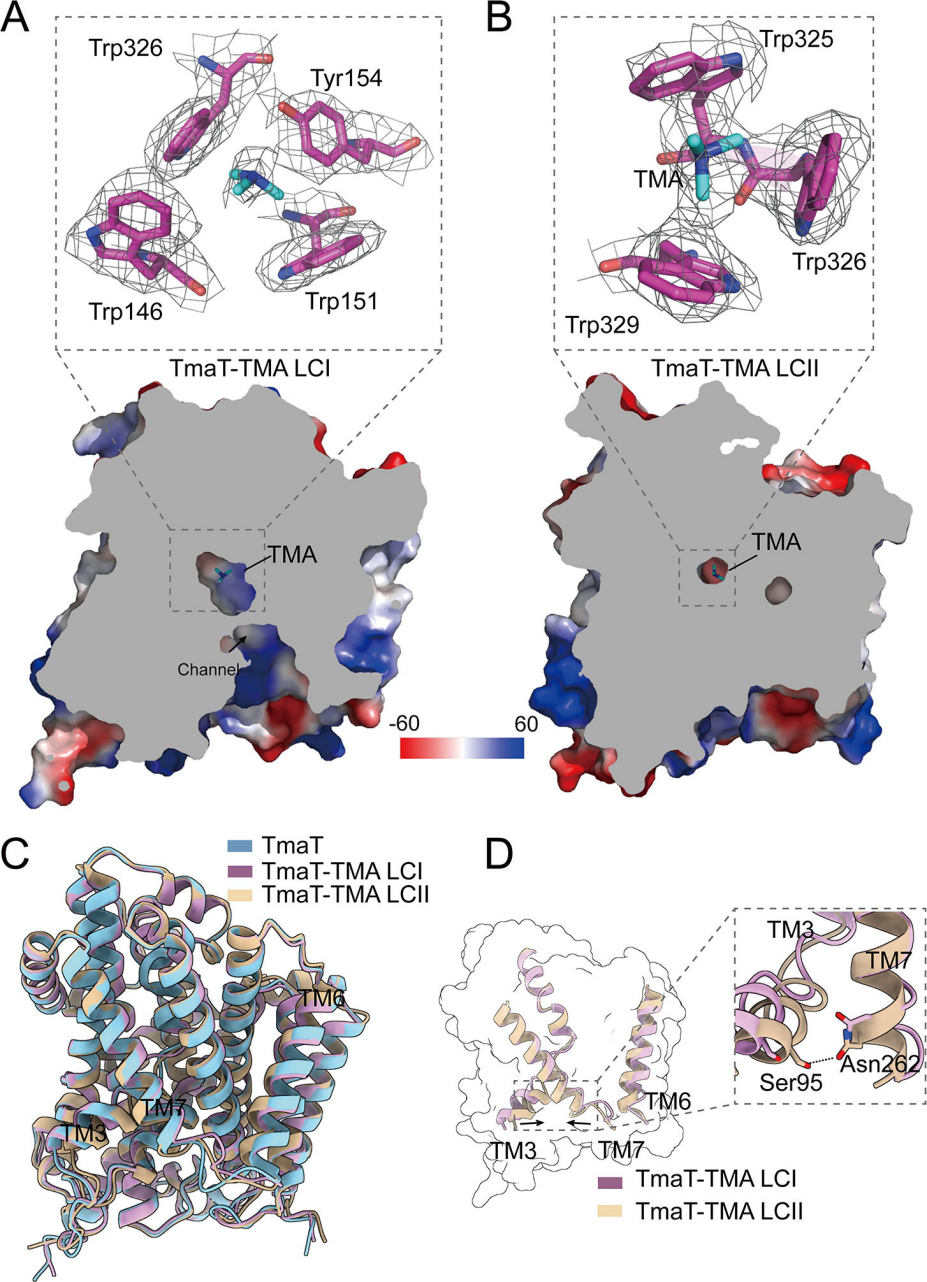
Comparison of the structures of TmaT and TmaT-TMA complexes. (**A**) Structural alignment of TmaT, TmaT-TMA LCI, and TmaT-TMA LCII. (**B**) Cross-sectional view of TmaT-TMA LCI. The cryo-EM density of TMA and the surrounding residues is contoured in gray at 1.8σ. The TMA molecule and the surrounding residues are shown as cyan and magenta sticks, respectively. (**C**) Structural alignment of TmaT-TMA LCI and TmaT-TMA LCII. (D) Cross-sectional view of TmaT-TMA LCII. The cryo-EM density of TMA and the surrounding residues is contoured in gray at 1.8σ. The TMA molecule and the surrounding residues are shown as cyan and magenta sticks, respectively.

To probe the transport mechanism of TmaT, we further solved two structures of TmaT-TMA complexes, TmaT-TMA LCI and TmaT-TMA LCII, in which TMA is located in different binding sites (Fig. S4 and S5; Table S2). In comparison to the electron densities of TmaT, additional electron densities at the potential TMA-binding sites were observed in the TmaT-TMA complexes, indicating the positions of TMA molecules (Fig. 3A and B). The TMA-binding site in TmaT-TMA LCI corresponds to the betaine-binding site I in BetP (31), while the TMA-binding site in the TmaT-TMA LCII corresponds to the betaine-binding site II in BetP (34). The overall structures of TmaT, TmaT-TMA LCI, and TmaT-TMA LCII monomers are similar (Fig. 3C), with the RMSD values ranging from 0.4 to 0.7 Å. Analysis of the TmaT-TMA LCI structure suggested that the TMA translocation channel between the transmembrane helices TM3 and TM7 is oriented toward the cytoplasm, forming an inwardly open conformation and allowing the passage of TMA (Fig. 3D). Upon comparing the structures of TmaT-TMA LCI and TmaT-TMA LCII, it is evident that the proximity of TM3 and TM7 resulted in the closure of the substrate channel (Fig. 3D). Structural analysis indicated that the newly formed hydrogen bond between residues Asn262 and Ser95 in TmaT-TMA LCII may play a crucial role in the closure of the substrate channel (Fig. 3D).

### TMA-binding sites

**Fig 4:**
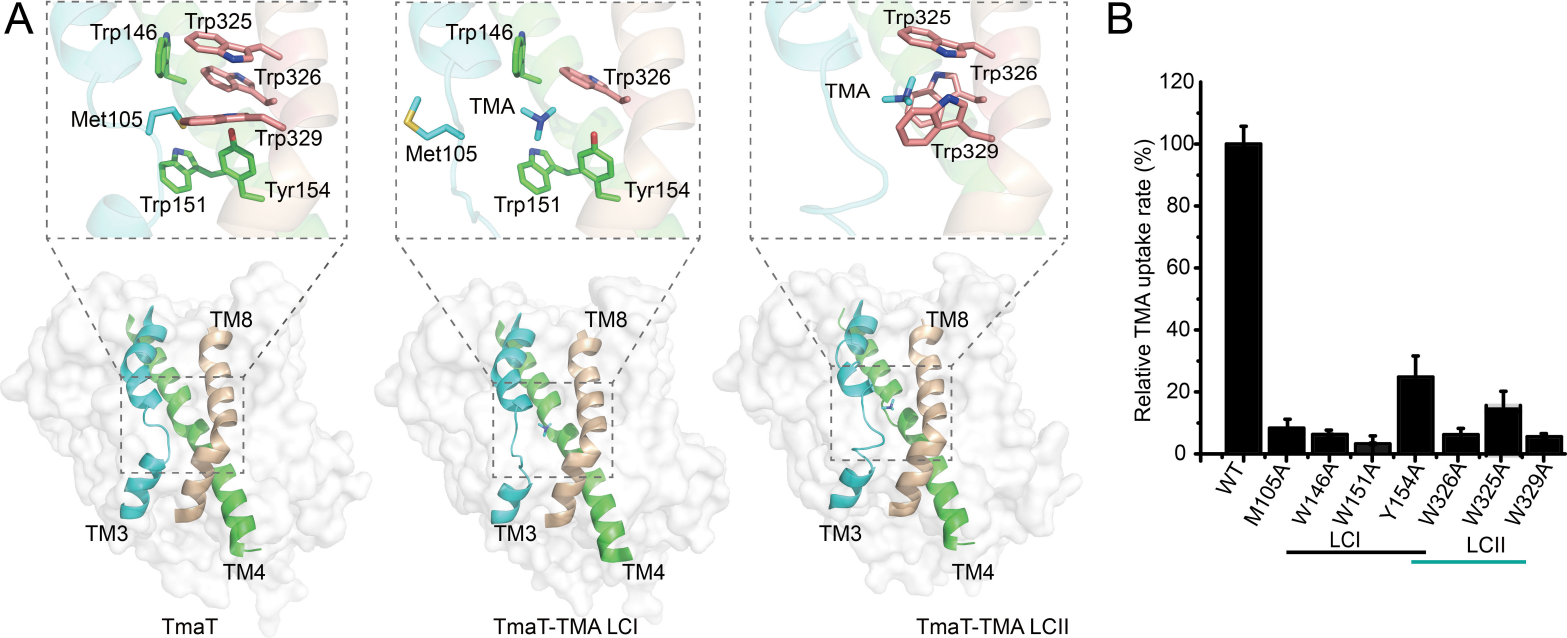
Analyses of the residues involved in TMA and sodium binding of TmaT. (**A**) Analyses of the aromatic boxes involved in binding TMA in the structures of TmaT, TmaT-TMA LCI, and TmaT-TMA LCII. Cartoon and surface representations of the structures in side view with the TM3 are colored in cyan, TM4 in green, and TM8 in wheat. Residues involved in TMA binding are shown as sticks. (**B**) Analyses of the TMA transport activities of TmaT and its mutants. The TMA transport activity of TmaT and its mutants was determined by GC with proteoliposomes. The TMA transport activity of WT TmaT was taken as 100%. The error bars represent standard deviation of triplicate experiments.

Analysis of the TmaT-TMA LCI structure indicated that when TMA is located at the substrate-binding site I (LCI), it is surrounded by four aromatic amino acid residues (Trp146, Trp151 and Tyr154 from TM4, and Trp326 from TM8), forming an aromatic box (Fig. 4A). Comparison of the TmaT and TmaT-TMA LCI structures revealed that the side chain of Met105 occupies the TMA-binding site when TMA is not bounded (Fig. 4A), suggesting an important role of Met105 in TMA recognition. In the structure of TmaT-TMA LCII, the TMA molecule is positioned at the second substrate-binding site (LCII), which is downstream of LCI within the substrate transport channel (Fig. 4A). The LCII is also composed by three aromatic residues (Trp325, Trp326, and Trp329 in TM8) (Fig. 4A). Comparison of the TmaT-TMA LCI and TmaT-TMA LCII structures revealed that when TMA enters the aromatic box at the LCII site, the side chain of Trp326 flips inward and acts as a lid to control the opening and closing of the aromatic box (Fig. 4A). To verify the functions of these residues, we generated site-directed mutations to these residues and measured the TMA transport activities of the mutants. The TMA transport activity of the M105A, W146A, W151A, Y154A, W325A, W326A, and W329A mutants was largely impaired (Fig. 4B), indicating the important roles of these residues in TMA transport. In BCCT transporters, the movement of substrates between different aromatic boxes serves as the foundation for the inward substrates transportation (30, 31).

### Molecular mechanism of TMA transport by TmaT

**Fig 5:**
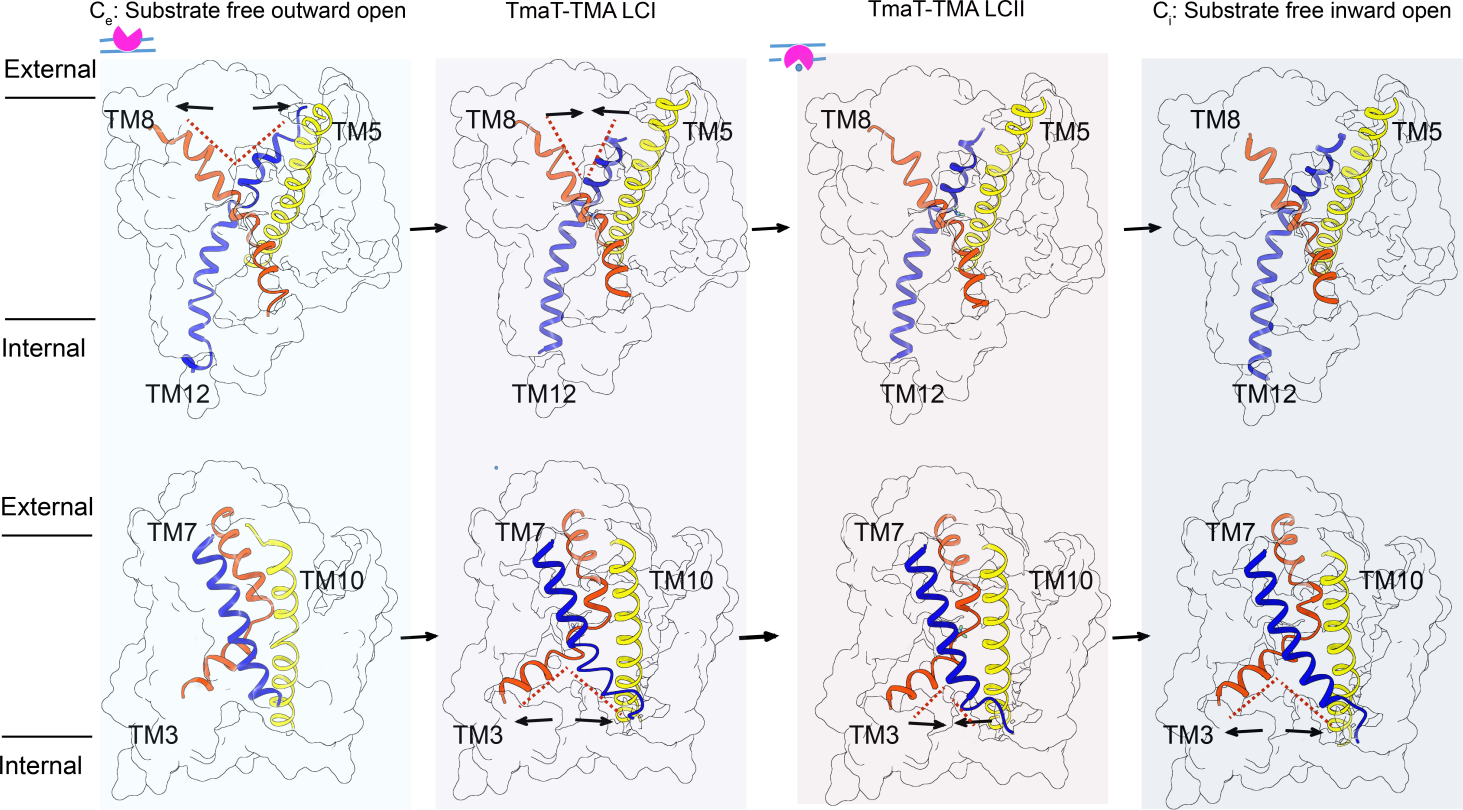
The proposed transport process of TMA by TmaT. The corresponding transmembrane helices in two inverted repeats are colored consistently.

To investigate the transport process of TMA by TmaT, we conducted molecular dynamic simulations using a phospholipid bilayer containing TmaT (Fig. S6). Upon reaching equilibrium (Fig. S6A), TmaT showed the substrate-free inward open (*C*_*i*_) conformational changes of the first principal component (contributing ~35% of the overall conformational changes) and the substrate-free outward open (*C*_*e*_) conformational changes of the second principal component (contributing ~5% of the overall conformational changes), which is also supported by cluster analysis (Fig. S6B). Therefore, in addition to TmaT-TMA LCI and TmaT-TMA LCII, two representative structures possessing *C*_*e*_ and *C*_*i*_ conformations were selected for further analysis (Fig. 5).

In the *C*_*e*_ conformation, the transport channel of TMA opens toward the extracellular side of the membrane due to the outward expansion of TM8 and TM12. In the TmaT-TMA LCI, the side chain of Met105 undergoes a conformational change, freeing up the binding site for TMA (Fig. 4A). In this conformation, TM8 and TM12 approach each other, while TM3 and TM7 get far away from each other, resulting in an inward open conformation. Analysis of the structures of TmaT-TMA LCI and TmaT-TMA LCII suggests that after the binding of TMA, the hydrogen bond formed between residues Ser95 and Asn262 may play a crucial role in the transition between these two conformations. The formation and dissociation of the hydrogen bond were also observed during the MD simulations with the TmaT-TMA LCII structure, supporting the structural observations (Fig. S6C and D). Additionally, the distances between TMA molecule and the side chains of residues Trp325, Trp326, and Trp329 are relatively steady (Fig. S6E), suggesting that the binding of TMA in location II is relatively stable. In the *C*_*i*_ conformation, the outward expansion of TM3 and TM7 allows the TMA transport channel to open toward the intracellular side of the membrane. The alternating opening of the substrate channel toward the intracellular and extracellular sides drives the transport of TMA molecules.

## DISCUSSION

In this study, we analyzed the three-dimensional structure of TmaT, which exhibits the transmembrane helix folding mode of the LeuT-fold (21, 35). Biochemical results suggested that TmaT is a TMA-specific transporter, which is in consistent with previous reports that transporters adopting the LeuT-fold exhibit strong substrate specificity (21, 35). The overall structure of TmaT closely resembles BetP (with an RMSD of 1.23 Å over 388 Cα atoms) and CaiT (with an RMSD of 2.12 Å over 412 Cα atoms) (Fig. S7A). However, they diverge in substrate recognition, as the side chain of Met105 in TmaT occupies the substrate-binding pockets during substrate binding, a characteristic not observed in the structures of BetP and CaiT (30, 31). The residue Met105 is located in the “GXGXG” motif in the discontinuous TM3. In the sodium/neurotransmitter symporter MhsT, a member of the LeuT-fold transporter, the flexible “GXGXG” motif has also been shown to modulate substrate recognition (36). It is striking that the glycine betaine transporters of the BCCT family possess a highly conserved “GXGXG” motif located in TM3, which may provide a crucial foundation for the binding of substrates (22, 37). However, TmaT did not bind glycine betaine (Table 1). Our results suggested two potential reasons contributing to the substrate specificity of TmaT: (i) TMA molecules are considerably smaller than glycine betaine, and the smaller aromatic box formed by TmaT during transportation may not accommodate larger molecules like glycine betaine (Fig. S7B and C). (ii) Under neutral conditions, TMA molecules remain uncharged compared to other small quaternary ammonium molecules, and the charges carried by these substrates themselves may influence the recognition and transportation process of TmaT. As an Na^+^/TMA symporter, Na^+^ is of vital importance for TMA transport. Sequence analysis indicated that TmaT contains two conserved Na^+^-binding sites (Na1 and Na2) (Fig. S8) that are identical to those identified in BetP (38). However, no electron density corresponding to Na^+^ was identified in any of the three determined TmaT structures, suggesting a relatively flexible binding of Na^+^.

**Fig 6:**
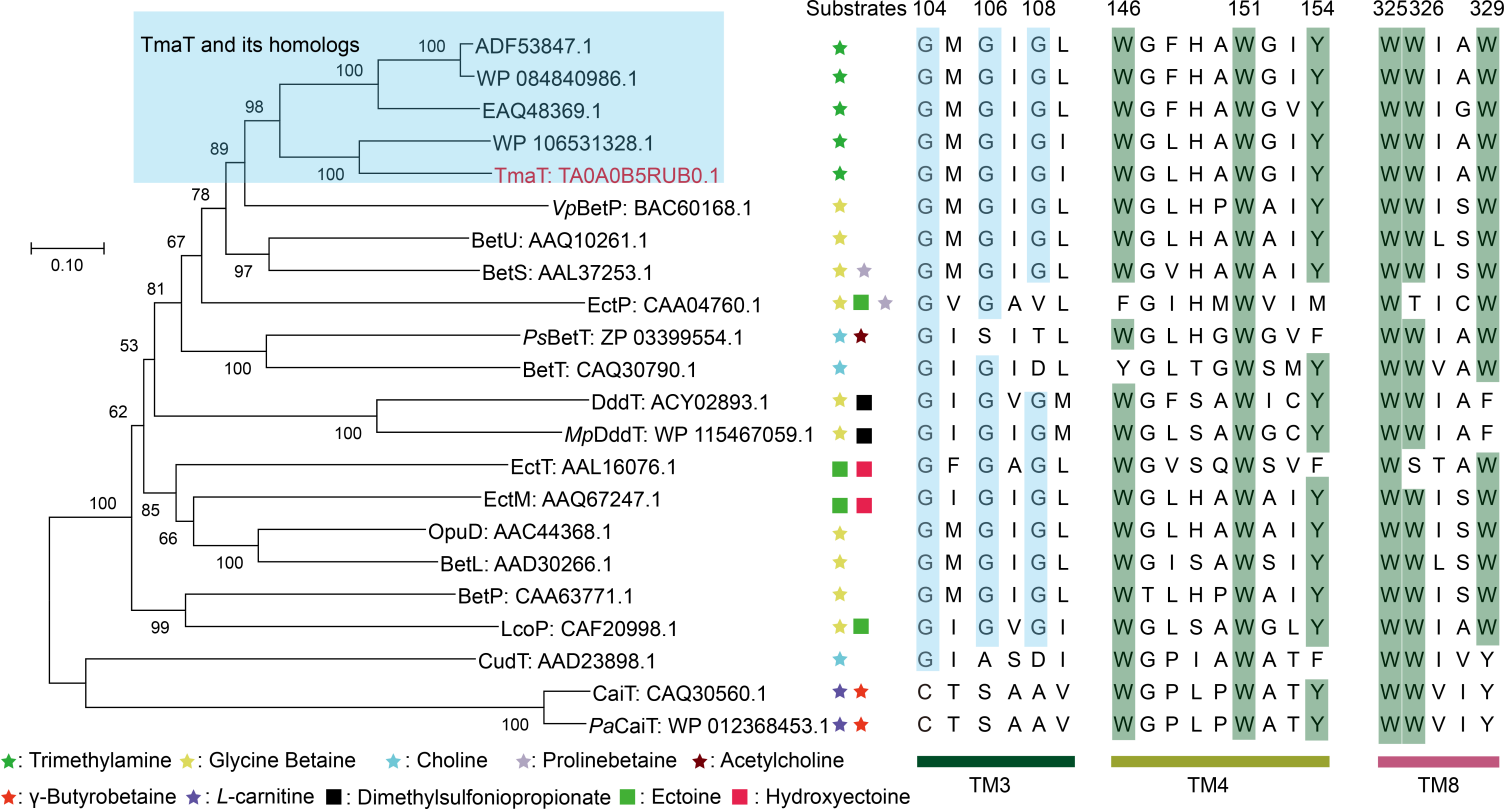
Phylogenetic analysis and sequences alignment of TmaT and other members from BCCT family. The glycine residues in the “GXGXG” motif are colored in blue. Residues involved in TMA binding are colored in green. TmaT homologs (sequence similarity: 51%–83%) and other BCCT carriers were extracted from the non-redundant protein sequence database.

In recent years, several transporters from the BCCT family that do not possess quaternary ammonium structures in their substrates have been identified (Table S1), such as DddT for transporting dimethylsulfoniopropionate (DMSP) and EctP for transporting ectoine (22, 39, 40). Phylogenetic analysis and multiple sequence alignments were performed between TmaT and other BCCT family members (Fig. 6). The alignment results revealed that the amino acid residues Trp151 and Trp325, which are involved in TMA binding in the TmaT protein, are highly conserved among all the BCCT transporters. The residues Trp146, Tyr154, Trp326, and Trp329, however, are only conserved in the transporters that transport molecules containing quaternary ammonium structures (Fig. 6). By conducting multiple sequence alignments of known functional BCCT family proteins, we can gain insights into the transport substrates of unknown functional BCCT family proteins. For instance, Phe329 may serve as a distinctive marker for the DMSP transporter (39), and Phe154 as a marker for choline transporter (22, 24). Instead, members of the conservative “GXGXG” carriers are energized by the sodium-coupled symporter (22, 23, 25, 39), with Asp108 representing a proton-coupled symporter (22, 24, 25, 28).

In summary, TMA is an important organic nitrogen compound widely distributed in nature. TmaT is a TMA-specific transporter belonging to the BCCT family. Our results suggested that TmaT is an Na^+^/TMA symporter. The TMA binding and transport mechanisms were also revealed based on structural and biochemical assays. The results of this study will significantly enhance our understanding of the TMA transport processes across biological membranes. Moreover, our findings expand the current knowledge on BCCT family transport proteins and deepen our understanding of Na^+^-coupled transport proteins.

## MATERIALS AND METHODS

### Bacterial strains and growth conditions

Strain *M. profundi* D25 was previously maintained in our lab and was cultured in 2216E medium at 25°C according to the provided protocol (http://www.dsmz.de/). *E. coli* strains DH5α and C43(DE3) were grown in lysogeny broth (LB) medium at 37°C.

### Gene cloning, point mutation, and protein expression and purification

The complete *tmaT* gene was amplified from the genome of *M. profundi* D25 by PCR using *FastPfu* DNA polymerase (TransGen Biotech, China). The amplified gene was then inserted into the pET-22b vector (Novagen, Germany) with a C-terminal 8× His tag. All of the site-directed mutations were performed with the QuikChange Mutagenesis Kit II (Agilent, America) and were verified by DNA sequencing. The TmaT protein and its mutants were expressed in *E. coli* C43(DE3). The cells were grown at 37°C in LB medium to an OD_600_ of 0.8–1.0 and then incubated at 16°C for 16 hours with 0.2 mM isopropyl β-d-1-thiogalactopyranoside as an inducer for recombinant protein expression. After induction, cells were harvested by centrifugation (10 min, 7,000 × *g*, 4°C) and resuspended in the lysis buffer (100 mM NaCl, 0.5% glycerol, 50 mM Tris-HCl, pH 7.8) to be disrupted by high-pressure cell cracker. Cell lysate was centrifuged at 15,000  × *g* at 4°C for 30 min to remove insoluble cell debris and then subjected to ultracentrifugation at 200,000 × *g*, 4°C for 60 min. Membrane pellets were resuspended in the solubilization buffer (1% [wt/vol] DDM, 250 mM NaCl, 15% [vol/vol] glycerol, 150 mM Tris-HCl, pH 7.8) for 12 h at 4°C and ultracentrifuged for 30 min, 4°C, 200,000  × *g* to remove insoluble material. The recombinant proteins were purified first with Ni^2+^ affinity column (GE Healthcare, America) and then with gel filtration on a Superdex-200 column (GE Healthcare, America) eluted with the elution buffer (0.02% [wt/vol] DDM, 100 mM NaCl, 10 mM Tris-HCl, pH 7.0).

### Microscale thermophoresis-binding assay

The binding affinities of compounds to TmaT were assessed using the NanoTemper Monolith NT.115 MST system. Purified TmaT was labeled using the Labeling Kit (RED-Tris-NTA 2nd Generation). Substrates were diluted to various concentrations (from 76.3 nM to 2.5 mM) and mixed with labeled TmaT at 22°C in the PBS-T buffer (1× PBS [pH 7.5], 0.02% [vol/vol] DDM, 250 mM NaCl, and 0.01% Tween-20). The mixture was then loaded into Monolith NT.115 Series capillaries (NanoTemper Technologies, Germany), and thermophoresis was performed using a Monolith NT.115 instrument (NanoTemper Technologies, Germany). Binding was measured with 40% LED power and “medium” MST power, with an optimized time setting (5 s Fluo, before; 30 s MST On; 5 s Fluo, after). Each assay was conducted with a minimum of three biological replicates, and the data were analyzed using Affinity Analysis (MST) v2.3 software.

### Transport assay

TMA transport mediated by TmaT was measured in *E. coli* polar lipid proteoliposomes. The proteins were first reconstituted into *E. coli* phospholipids (41), and then substrate transport assay in proteoliposomes was performed by adding TMA to a final concentration of 100 mM and then incubated at 4°C for 2 hours. After the incubation, the samples were ultracentrifuged at 4°C and 600,000 × *g* for 15 min, and the pellets were resuspended in the buffer containing 10 mM Tris-HCl (pH 7.0) and 100 mM NaCl. This process was repeated twice to remove any TMA that did not transport into the membrane and the TMA adhered to the surface of proteoliposomes. Following this, the samples were promptly transferred to a sealed gas-phase vial, and 1 mol/L NaOH was added to disrupt the proteoliposomes, thereby releasing the TMA that had been transported into the proteoliposomes. The uptake of TMA by proteoliposomes was then assayed on a gas chromatograph (Shimadzu, Japan). The detection was performed using a Carbowax Amine capillary column (30 m × 0.53 mm × 1.0 µm) and a Flame Ionization Detector. TMA standard solutions ranging from 2 to 40 µM were analyzed under the same conditions to generate a standard curve for TMA. A five-point calibration curve of TMA standards was used.

### Electron microscopy sample preparation, data acquisition, and image processing

Purified TmaT (4 µL at a concentration of approximately 8  mg/mL) was applied to glow-discharged holey carbon grids (200 mesh, copper). Grids were blotted for 2 s at 100% humidity and 8°C with a force level of −2 and then flash frozen in liquid ethane cooled by liquid nitrogen using a Vitrobot Mark IV (FEI, America). Grids were imaged with a 300-keV Titan Krios (FEI, America) electron microscope, equipped with a K3 BioQuantum electron counting direct detection camera (Gatan, America). All cryo-EM movies were automatically recorded using EPU (Thermo Fisher, America) at a nominal magnification of ×81,000, corresponding to a calibrated physical pixel size of 0.53 Å with the energy filter slit set to 20 eV (42). The defocus range was set between −1.2 and −2.2  µm, and the total dose for each stack was about 50 e^−^/Å^2^. To obtain the TmaT-TMA complex (TmaT-TMA LCI), 2 mM TMA was added into TmaT, and the mixture was incubated at 4°C for 30 min. To prepare the TmaT-TMA LCII complex, 5 mM TMA was added into TmaT, and then the mixture was incubated at room temperature for 1 hour. The subsequent data collections were performed in accordance with the procedures described for TmaT. The data processing details are summarized in Table S2.

For the TmaT structures, we collected 5,041 movie stacks which were motion corrected using MotionCor2.1 with dose weighting (43). CTFFIND4 was used to estimate the contrast transfer function (CTF) parameters for each movie (44). Next, 3,207,653 particles were auto-picked from all movie stacks and 2 rounds of 2D classifications were performed to exclude noise and other bad particles using cryoSPARC (45). Furthermore, 310,834 particles from qualified 2D averages were selected for further 3D analysis. Ahead of 3D classification, a round of refinement was applied to remove duplicated particles on the whole particle sets using RELION-3 (46). After 3D classification, the remaining 185,911 particles were measured in 3D refinement, CTF refinement, and 3D non-uniform refinement to generate a resolution map at 3.05  Å with a *B* factor of −127.4  Å^2^. A similar data processing strategy was employed for TmaT-TMA LCI and TmaT-TMA LCII. Specifically, 2,217,332 and 4,918,407 particles were extracted from 3,616 and 5,670 micrographs, respectively. After conducting 3D classification, 225,961 and 148,358 particles remained, resulting in 3D reconstructions with resolutions of 2.76 and 3.09 Å, respectively, as determined by the gold-standard Fourier shell correlation criterion. Simplified flowcharts for data processing of TmaT, TmaT-TMA LCI, and TmaT-TMA LCII are summarized in Fig. S2, S4, and S5.

### Model building and refinement

The crystal structure of BetP from *Corynebacterium glutamicum* (PDB: 2WIT) was fitted into the electron density map of TmaT at 3.05 Å using UCSF ChimeraX 1.4 (47). The model of TmaT fitted into the cryo-EM densities using Coot (48). The structure was refined using Phenix with phenix.real_space_refine (49), and the statistics of the refinement are listed in Table S2. All figures related to the structures were generated using PyMoL or UCSF ChimeraX 1.4 (47, 50).

### MD simulations

The lipid bilayer structures consisting of palmitoyl oleoyl phosphatidyl-glycerol with TmaT or TmaT-TMA LCII inserted in the center were generated by CHARMM-GUI server (51). A 500-ns molecular dynamics simulation of the entire system was performed by using GROMACS 2024.2 (52), with the Amberff99SB-ILDN forcefield adopted (53). The simulation was conducted under the NPT ensemble with periodic boundary conditions and a time step of 2 fs. The temperature of the system was kept at 298 K using the Nose-Hoover method, and the pressure was kept at 1 bar using the Parrinello-Rahman method. All MD simulations were conducted in triplicate to ensure the reliability and robustness of the results. According to the backbone-atom RMSD plot, trajectories which reached the equilibrium state (300–500 ns) were used for further analysis.

## Data Availability

The cryo-EM map and atomic coordinates have been deposited in the PDB and the Electron Microscopy Data Bank (EMDB) under accession code 8ZW8 and EMD-60519 for TmaT, 8ZXK and EMD-60542 for TmaT-TMA LCI, and 8ZXP and EMD-60548 for TmaT-TMA LCII. All MD simulation data are available upon request.

## SUPPLEMENTAL MATERIAL

Supplemental material mbio.01914-24-s0001.docx Fig. S1 to S8; Tables S1 and S2.

ASM does not own the copyrights to Supplemental Material that may be linked to, or accessed through, an article. The authors have granted ASM a non-exclusive, world-wide license to publish the Supplemental Material files. Please contact the corresponding author directly for reuse.
