# Supplementary material for: Structural basis of a microbial trimethylamine transporter
Source: mBio. 2024 Nov 22;16(1):e01914-24. doi: 10.1128/mbio.01914-24 (PMC11708041; doi:10.1128/mbio.01914-24)
Supplement: Supplemental material — Fig. S1 to S8; Tables S1 and S2. [file mbio.01914-24-s0001.docx]

**Structural basis of a microbial trimethylamine transporter**

Chao Gao^1,2,3,4#^, Hai-Tao Ding^5#^, Kang Li^2,4#^, Hai-Yan Cao^2,4^, Ning Wang^1,2,4^, Zeng-Tian Gu^1^, Qing Wang^1^, Mei-Ling Sun^2,4^, Xiu-Lan Chen^1,3,4^, Yin Chen^2,6^, Yu-Zhong Zhang^1,2,3,4^, Hui-Hui Fu^2,4^* and Chun-Yang Li^2,4^*

^1^State Key Laboratory of Microbial Technology, Marine Biotechnology Research Center, Shandong University, Qingdao, China.

^2^MOE Key Laboratory of Evolution and Marine Biodiversity, Frontiers Science Center for Deep Ocean Multispheres and Earth System & College of Marine Life Sciences, Ocean University of China, Qingdao, China.

^3^Laboratory for Marine Biology and Biotechnology, Qingdao Marine Science and Technology Center, Qingdao, China.

^4^Joint Research Center for Marine Microbial Science and Technology, Shandong University and Ocean University of China, Qingdao, China.

^5^Antarctic Great Wall Ecology National Observation and Research Station, Polar Research Institute of China, Ministry of Natural Resources, Shanghai, China.

^6^School of Life Sciences, University of Warwick, Coventry, UK.

^#^These authors contributed equally: Chao Gao, Hai-Tao Ding, Kang Li.

*Corresponding author: Hui-Hui Fu, fuhuihui@ouc.edu.cn; Chun-Yang Li, lcy@ouc.edu.cn.

**This file includes:**

Figures S1 to S8

Tables S1 to S2

**
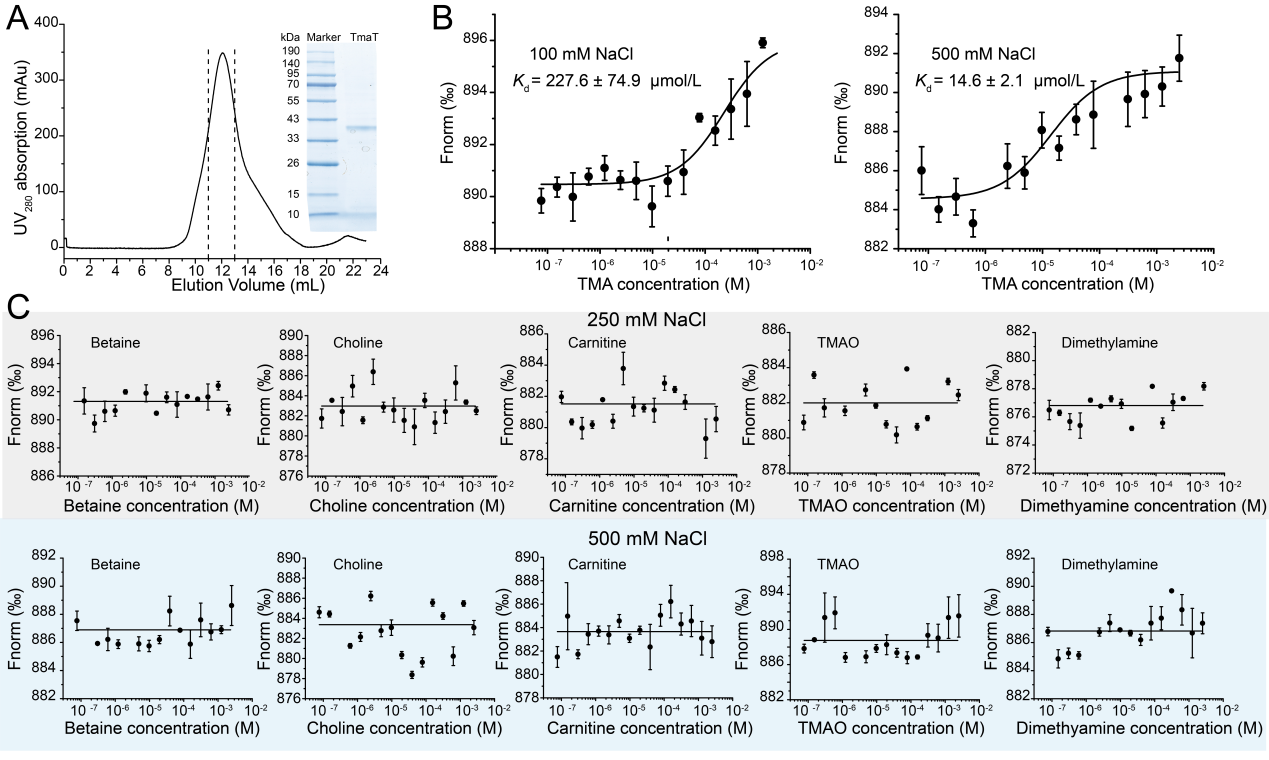
**

**FIG S1** Purification and binding affinity analysis of the recombinant TmaT. (**A**) Gel filtration and SDS-PAGE analyses of the recombinant TmaT. (**B**) MST analysis of the TMA binding activity of TmaT with different NaCl concentrations. (**C**) MST analysis of the binding activities of TmaT towards different substrates with 250 mM NaCl (gray background) or 500 mM NaCl (blue background) in solution. The y-axis represents the normalized fluorescence (Fnorm), and the error bars represent standard deviation from triplicate experiments.


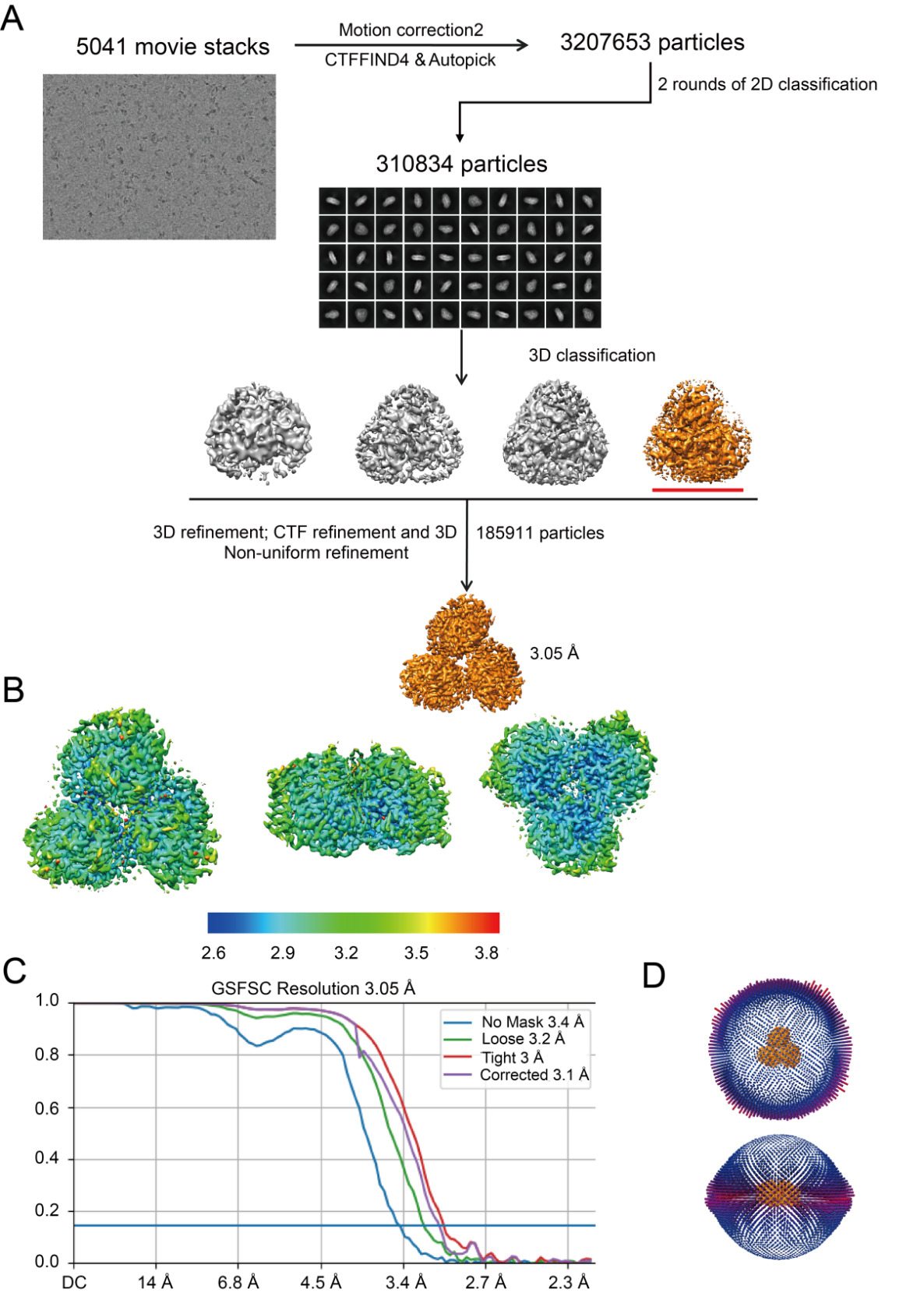


**FIG S2** Flowchart for cryo-EM single-particle data processing of TmaT. (**A**) A micrograph of the single particles after drift correction and dose-weighting, 2D classifications, 3D classifications and selections. (**B**) Cryo-EM map colored by local resolutions calculated using RELION 3.0. (**C**) Gold-standard Fourier shell correlation (FSC) curves of the final EM maps. (**D**) Angular distribution of the cryo-EM particles included in the final 3D reconstruction.


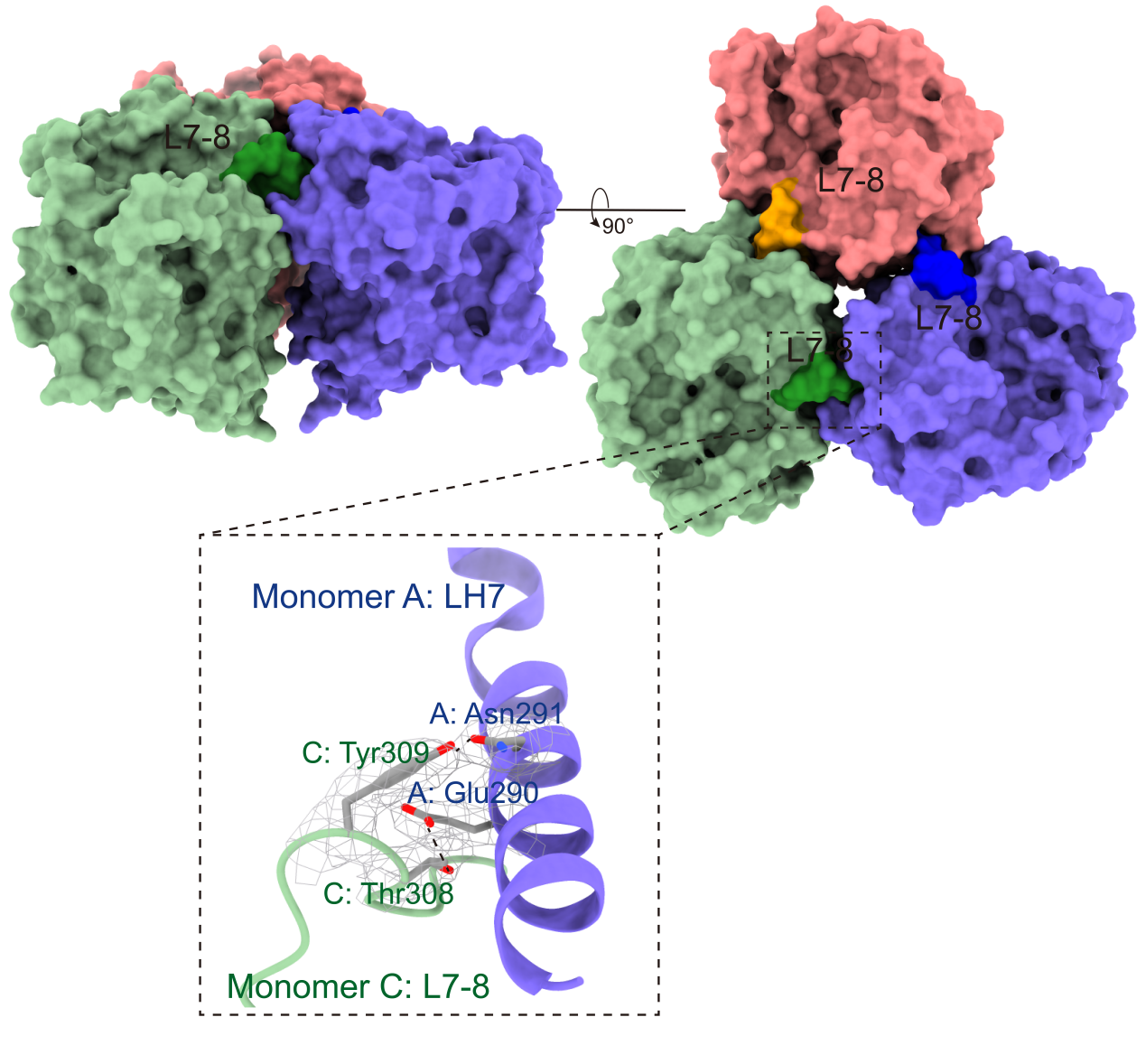


**FIG S3** The overall structure of the TmaT trimer with the top view and side view. Individual monomers are colored in blue, red and green, respectively.


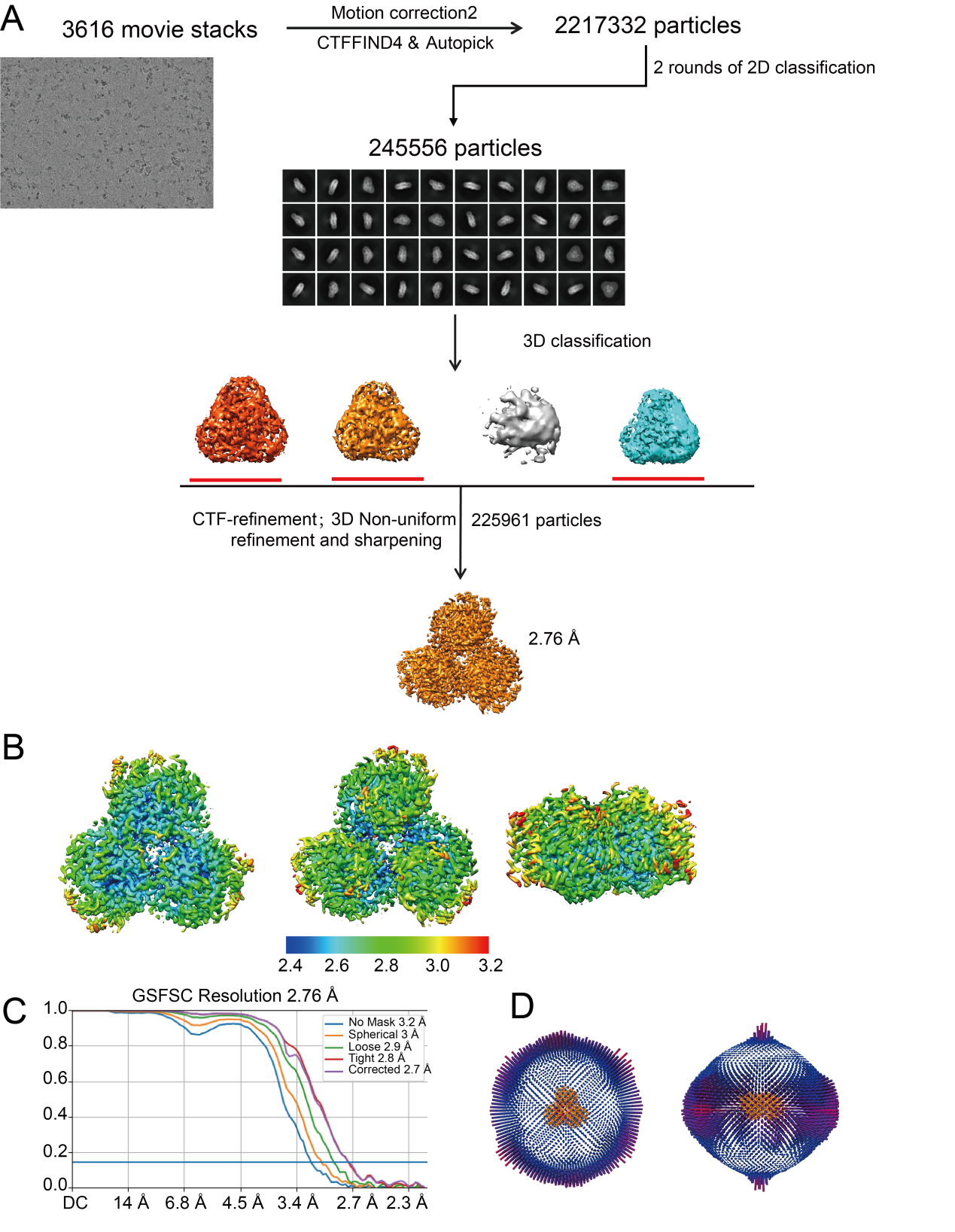


**FIG S4** Flowchart for cryo-EM single-particle data processing of TmaT-TMA LCⅠ. (**A**) A micrograph of the single particles after drift correction and dose-weighting, 2D classifications, 3D classifications and selections. (**B**) Cryo-EM map colored by local resolutions calculated using RELION 3.0. (**C**) FSC curves of the final EM maps. (**D**) Angular distribution of the cryo-EM particles included in the final 3D reconstruction.

**
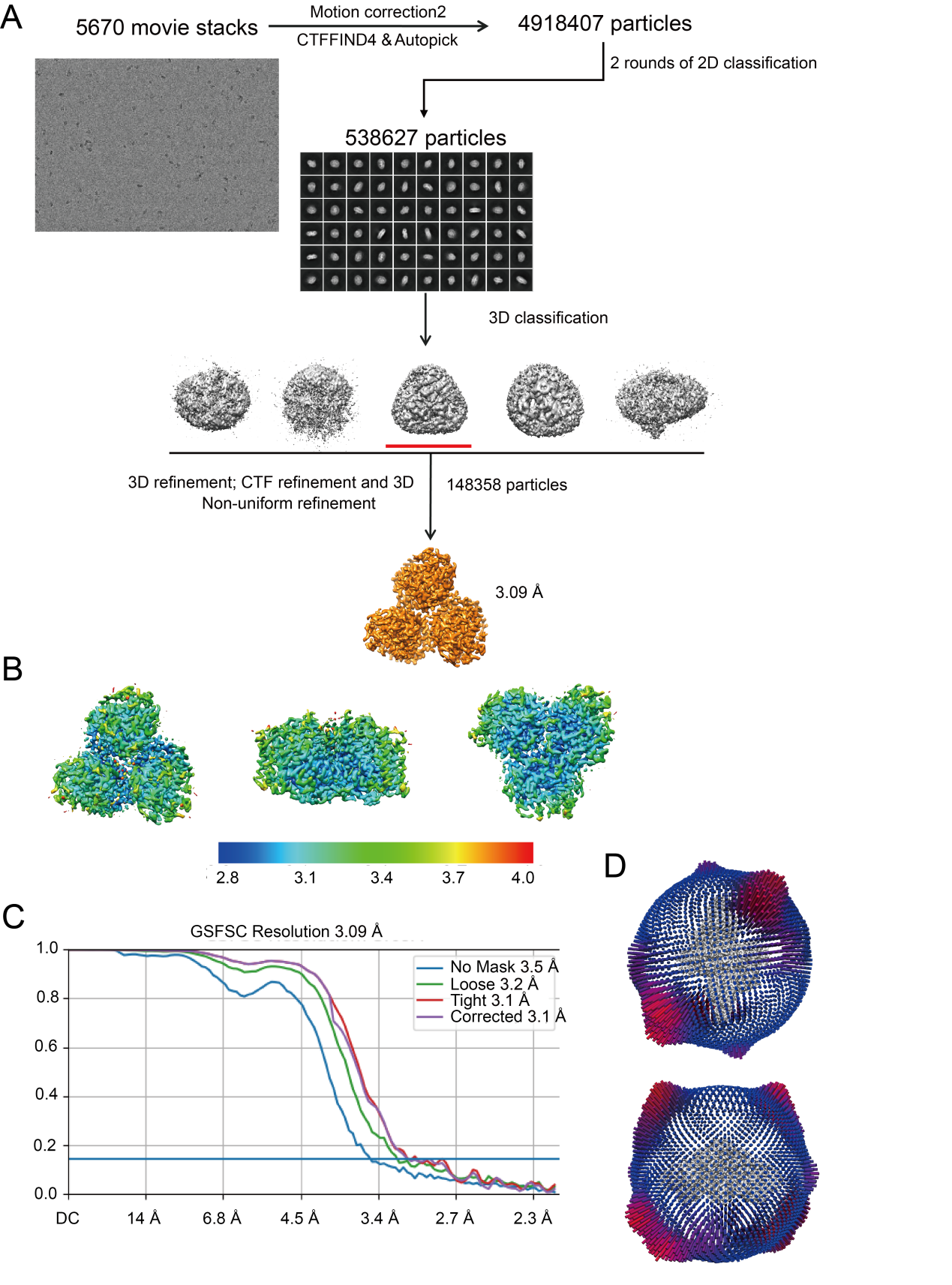
FIG S5** Flowchart for cryo-EM single-particle data processing of TmaT-TMA LCⅡ. (**A**) A micrograph of the single particles after drift correction and dose-weighting, 2D classifications, 3D classifications and selections. (**B**) Cryo-EM map colored by local resolutions calculated using RELION 3.0. (**C**) FSC curves of the final EM maps. (**D**) Angular distribution of the cryo-EM particles included in the final 3D reconstruction.


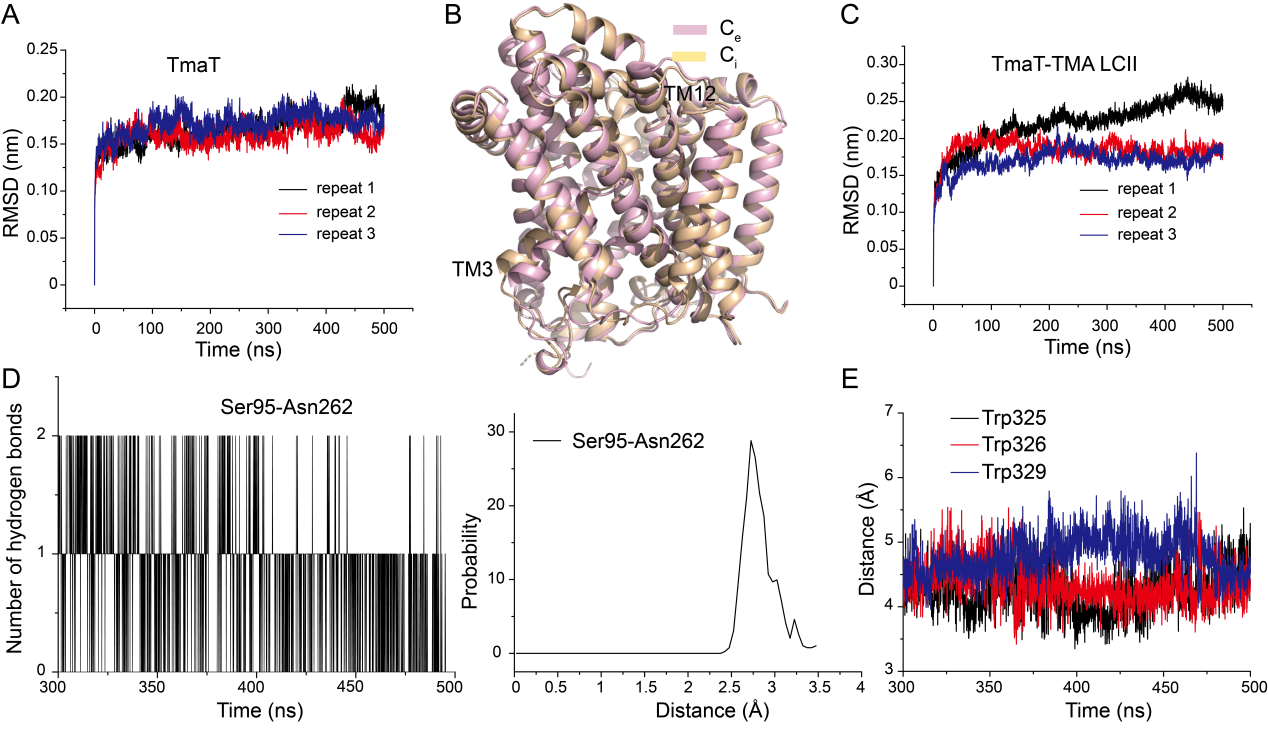


**FIG S6** MD simulations analyses of TmaT and TmaT-TMA LCⅡ. (**A**) RMSD of the backbone atoms of the TmaT structure during the 500 ns MD simulations. (**B**) TmaT structures at the C_e_ (colored in pink) and C_i_ (colored in wheat) conformations identified through cluster analysis. (**C**) RMSD of the backbone atoms of the TmaT-TMA LCⅡ structure during the 500 ns MD simulations. (**D**) Analysis of the hydrogen bond interactions between Ser95 and Asn262 in the TmaT-TMA LCⅡ MD simulations under the equilibrium states (300-500 ns). (**E**) Analysis of distances between the nitrogen atom of TMA and the side chains of residues Trp325, Trp326 and Trp329 in the TmaT-TMA LCⅡ MD simulations under the equilibrium states (300-500 ns).


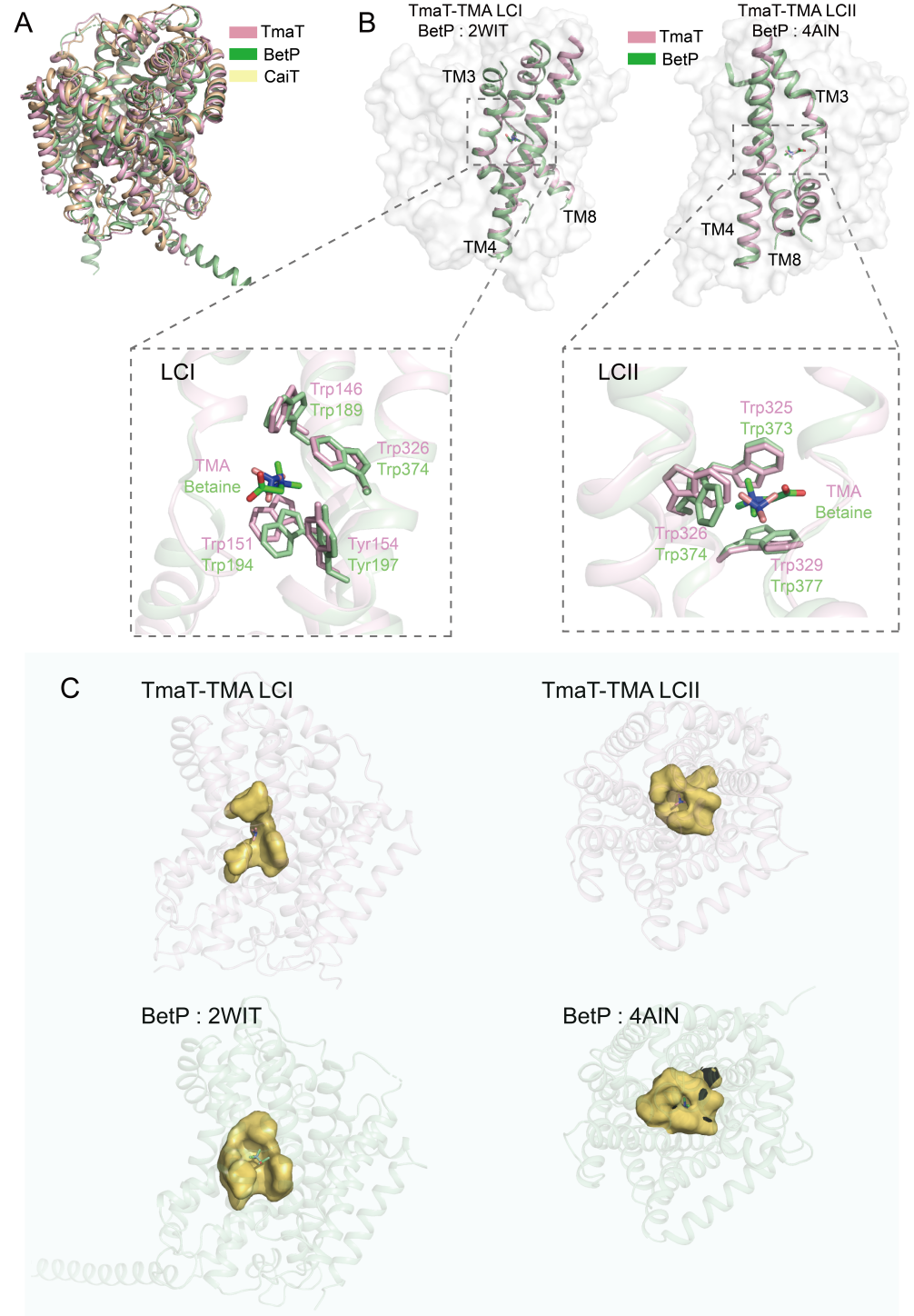


**FIG S7** Structural comparison of TmaT and other BCCT transporters. (**A**) Superposition of structures of TmaT, BetP (PDB code: 2WIT) and CaiT (PDB code: 3HFX). (**B**) Analysis of residues involved in substrate binding in TmaT and BetP. The residues in TmaT and BetP are shown in light pink and pale green sticks, respectively. (**C**) Comparison of the substrate binding pockets of TmaT and BetP. The binding pockets are colored in orange.


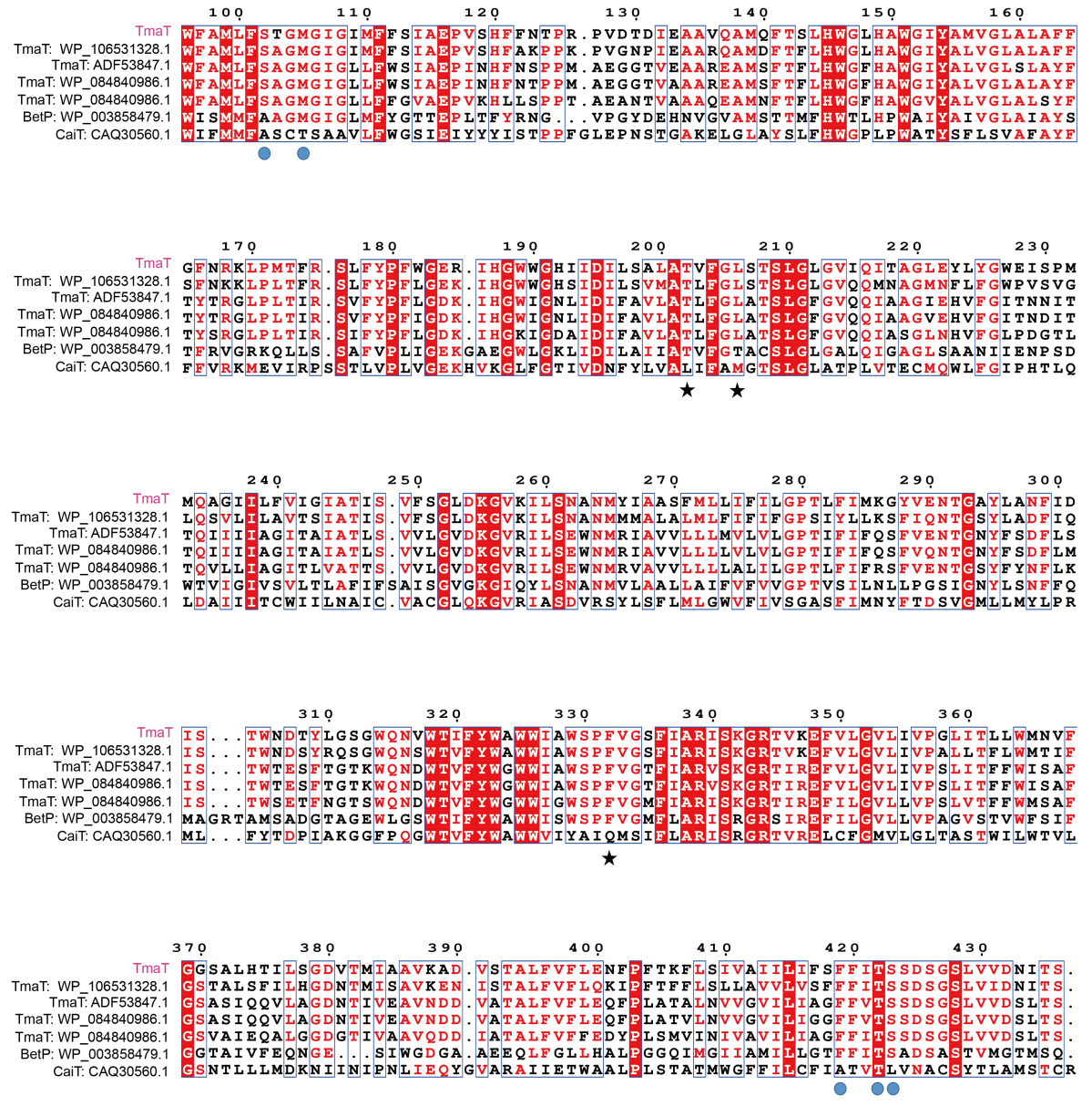


**FIG S8** Sequence alignment of TmaT homologs with BetP and CaiT. Residues involved in binding Na1 and Na2 are highlighted with blue circles and black stars, respectively.

**TABLE S1** Secondary active transporters in the BCCT family.

| **Transporters** | **Substrates** | **Force/direction*^a^*** | **Sequence identity to TmaT (%)** | **Total number (aa)** | **C-terminal domain (aa)** | **References** |
| --- | --- | --- | --- | --- | --- | --- |
| TmaT | TMA | Unknown | 100 | 527 | 45 | (Qin et al., 2021) |
| BetP | GB | Na^+^/solute symporter | 35 | 595 | 50 | (Ressl et al., 2009) |
| OpuD | GB | Na^+^/solute symporter | 40 | 512 | 24 | (Kappes et al., 1996) |
| BetL | GB | Na^+^/solute symporter | 40 | 507 | 20 | (Sleator et al., 1999) |
| BetH | GB | Na^+^/solute symporter | 35 | 505 | 18 | (Lu et al., 2004) |
| ButA | GB | Na^+^/solute symporter | 47 | 607 | 70 | (Baliarda et al., 2003) |
| *Vc*OpuD | GB | Na^+^/solute symporter | 43 | 539 | 15 | (Naughton et al., 2009) |
| BetU | GB | Na^+^/solute symporter | 45 | 667 | 162 | (Ly et al., 2004) |
| BetS | GB/PB | Na^+^/solute symporter | 45 | 706 | 167 | (Boscari et al., 2002) |
| DddT | GB/DMSP | Na^+^/solute symporter | 31 | 550 | 17 | (Todd et al., 2010) |
| LcoP | GB/E | Na^+^/solute symporter | 37 | 630 | 96 | (Steger et al., 2004) |
| EctT | E/HE | Na^+^/solute symporter | 32 | 501 | 11 | (Kuhlmann et al., 2011) |
| EctM | E/HE | Na^+^/solute symporter | 35 | 493 | 25 | (Vermeulen et al., 2004) |
| EctP | GB/E/PB | Na^+^/solute symporter | 38 | 615 | 102 | (Weinand et al., 2007) |
| *Ps*BetT | Ch/ACh | H^+^/solute symporter | 45 | 664 | 160 | (Chen and Beattie, 2008) |
| BetT | Ch | H^+^/solute symporter | 38 | 677 | 175 | (Lamark et al., 1991) |
| CudT | Ch | H^+^/solute symporter | 32 | 540 | 36 | (Rosenstein et al., 1999) |
| CaiT | CT/γ-BB | Substrate/product antiporter | 27 | 504 | 5 | (Schulze et al., 2010) |
| *Pm*CaiT | CT/γ-BB | Substrate/product antiporter | 28 | 514 | 10 | (Schulze et al., 2010) |

*^a^* The types of functionally characterized microbial transporters for compatible solutes.

TMA: Trimethylamine; GB: Glycine Betaine; PB: Prolinebetaine; DMSP: Dimethylsulfoniopropionate; E: Ectoine; HE: Hydroxyectoine; Ch: Choline; ACh: Acetylcholine; CT: *L*-carnitine; γ-BB: γ-Butyrobetaine.

**TABLE S2** Data collection, processing and refinement statistics of TmaT, TmaT-TMA LCⅠ and TmaT-TMA LCⅡ.

| **Parameters** | TmaT | TmaT-TMA LCⅠ | TmaT-TMA LCⅡ |
| --- | --- | --- | --- |
| **Data collection and processing** |  |  |  |
| Microscope | Titan | | |
| Magnification | 81000 | | |
| Voltage (kV) | 300 | | |
| Electron exposure (e-/Å2) | 53.87 | 51.48 | 50.23 |
| Camera | Gatan K3 | | |
| Defocus range (μm) | -1.2 ~ -2.2 | -1.5 ~ -2.5 | -1.2 ~ -2.2 |
| Pixel size (Å) | 0.53 | | |
| Symmetry imposed | C3 | | |
| Movies collected | 5041 | 3616 | 5670 |
| Final particle images (no.) | 185911 | 225961 | 148358 |
| Map resolution (Å) | 3.05 | 2.76 | 3.09 |
| FSC threshold | 0.143 | | |
| Sharpening *B*-factor (Å^2^) | -127.4 | -106 | -125.8 |
| Software used to process data | cryoSPARC v3.3.1 | | |
| **Refinement** |  |  |  |
| Initial model used (PDB code) | 2WIT | - | - |
| Non-hydrogen atoms | 11652 | 11653 | 11586 |
| Protein residues | 1491 | 1490 | 1482 |
| Ligands | - | TMA: 3 | TMA: 3 |
| *B*-factor (Å^2^) |  |  |  |
| Protein | 61.52 | 56.30 | 87.88 |
| Ligand | - | 44.23 | 57.24 |
| R.m.s. deviations |  |  |  |
| Bond lengths (Å) | 0.003 | 0.005 | 0.005 |
| Bond angles (°) | 0.594 | 0.959 | 0.977 |
| Validation |  |  |  |
| MolProbity score | 1.73 | 2.39 | 1.61 |
| Clash score | 8.99 | 8.74 | 4.76 |
| Poor rotamers (%) | 0.65 | 0.16 | 0.41 |
| Map VS model |  |  |  |
| CC_mask_ | 0.82 | 0.82 | 0.80 |
| CC_volume_ | 0.78 | 0.77 | 0.79 |
| CC_peak_ | 0.61 | 0.61 | 0.55 |
| Ramachandran plot (%) |  |  |  |
| Favored | 96.30 | 95.28 | 94.78 |
| Allowed | 3.70 | 4.72 | 5.22 |
| Disallowed | 0 | 0 | 0 |
| PDB access code | 8ZW8 | 8ZXK | 8ZXP |
| EMDB access code | 60519 | 60542 | 60548 |
